# Erythropoietin and Ferulic Acid Loaded on Fe_3_O_4_ Nanoparticles Exert Therapeutic Effect against Acute Kidney Injury Induced by Cisplatin Via Bcl-2/Bax, IL-6, TGF-β, and GPX-4 Mechanisms

**DOI:** 10.1007/s12010-026-05599-9

**Published:** 2026-02-21

**Authors:** Lamiaa A. A. Barakat, Aya Ashour Aref, Salma M. Khirallah

**Affiliations:** 1 Chemistry Department (Biochemistry Division), Faculty of Science, Port Said University, Port Said, 42526 Egypt; 2 Faculty of Applied Health Sciences, East Port Said National University, Port Said, Egypt

**Keywords:** Cisplatin, Ferulic acid, Erythropoietin, Fe_3_O_4_-NPs

## Abstract

Background: Acute kidney damage occurs in 30%–46% of patients receiving cisplatin chemotherapy; nevertheless, effective preventive and treatment strategies are insufficient. Ferulic acid, despite its potent antioxidant and anti-inflammatory properties, exhibits from poor aqueous solubility, rapid metabolism, and low bioavailability, which significantly limit its therapeutic efficiency in vivo when administered in its free form. Therefore, an effective delivery system was required to overcome these limitations, enhance FA stability, and prolong its biological residence time in renal tissue. Fe₃O₄ nanoparticles provided an appropriate solution to this challenge by coating it to a core-shell system of Fe₃O₄@C nanoparticles, which were selected not only as a carrier but also to enhance FA delivery, maintaining its therapeutic activity, and providing superior nephroprotection. Objectives: The present research demonstrates the therapeutic efficacy of Erythropoietin (EPO), magnetic nanoparticles (Fe_3_O_4_-NPs), Ferulic acid (FA), and FA-loaded Fe_3_O_4_-NPs@C against AKI induced by cisplatin. Methods: Forty-two rats were divided into seven groups (*n* = 6). Normal control, cisplatin (6 mg/kg), cisplatin + EPO (1000 IU/kg, intraperitoneally), cisplatin + FA (50 mg/kg, orally), cisplatin + Fe_3_O_4_-NPs (5 mg/kg, intraperitoneally), combination group (cisplatin, EPO, FA, and Fe_3_O_4_-NPs), and cisplatin + FA/Fe_3_O_4_-NPs@C (5 mg/kg, intraperitoneally). Euthanisation occurred after 7 days of administration. Results: Cisplatin-induced nephrotoxicity significantly elevated renal markers compared to the normal group (*p* ≤ 0.05), with creatinine reaching 0.93 mg/dL and urea 116.88 mg/dL. Treatment with ferulic acid (FA), Fe_3_O_4_-NPs, and erythropoietin (EPO) reduced these levels. At the same time, their combination and FA/Fe_3_O_4_-NPs@C further significantly decreased creatinine and urea levels (*p* ≤ 0.05), thereby restoring renal function and elevating antioxidants (catalase, glutathione reductase, superoxide dismutase). Notably, GPX-4 levels markedly increased (*p* ≤ 0.05) in the FA/Fe_3_O_4_-NPs@C group, confirming ferroptosis inhibition via upregulated ferritin and GSH; Bcl-2 expression rose while Bax declined (*p* ≤ 0.05), indicating anti-apoptotic effects. These biochemical changes were corroborated by histopathological and immunohistochemical findings, with reduced inflammation (downregulated IL-6) and fibrosis (downregulated TGF-β). Conclusion: FA/Fe_3_O_4_-NPs@C protects against nephrotoxicity of cisplatin by restoring renal antioxidant defenses, inhibiting inflammation, ferroptosis, and apoptosis, thereby enhancing renal function and recovery.

**Supplementary Information:**

The online version contains supplementary material available at 10.1007/s12010-026-05599-9.

## Introduction

A platinum-based chemotherapy medication called cisplatin is used to treat various kinds of malignancies, including testicular tumors, bladder cancer, and ovarian cancer. About 20–35% of patients taking cisplatin have developed complications such as nephrotoxicity leading to acute kidney injury (AKI), and the elevation of creatinine and urea levels. Besides, reducing red blood cells leads to anemia and weight loss [1, 2]. Cisplatin administration also develops AKI due to the drug’s tubular accumulation causing many cytotoxic mechanisms presented in DNA damage by forming purine cross-links in deoxyribonucleic acid, as well as the dysfunction of cellular organelles, mainly mitochondria and endoplasmic reticulum, which then activates apoptotic pathways, resulting in an increase of reactive oxygen species (ROS), oxidative stress, inflammation, and mitochondrial malfunction with no universally effective pharmaceutical nephroprotective method currently established [1, 3, 4].

The glycoprotein hormone erythropoietin (EPO) is mainly produced in renal cortex. Hypoxic conditions influence its expression as it is highly expressed when anemia develops and oxygen content reduces [5]. Both anemia and AKI are associated and linked with mortality in several populations, such as patients with chronic heart failure [6]. rHuEPO, or Epoetin, it has a comparable amino acid sequence to human endogenous EPO so it promotes erythropoiesis, increasing hemoglobin levels and lowering the need for transfusions, especially for cancer patients after chemotherapy and radiotherapy, such as cisplatin [5]. Recent research has shown that epoetin not only causes erythropoiesis but also reduces oxidative stress by activating the p13k/Akt pathway and has anti-inflammatory properties via lowering the release of cytokines that promote inflammation by stimulating the nuclear factor-kappa B (NF-κB) and reduction of proinflammatory cytokines, thereby regulating the expression of mitochondrial Bax/Bcl-2 [5, 6]. Erythropoietin (EPO) has been identified as a renoprotective drug in experimental cisplatin-induced nephrotoxicity, mitigating functional impairment and structural tubular damage via anti-apoptotic and anti-oxidative pathways [4, 7].

Ferulic acid (FA) is also a derivative of cinnamic acid. It exhibits therapeutic efficacy against numerous diseases. It comes from medical plants and natural sources such as oranges, tomatoes, carrots, sweet corn, spinach, grapes, and whole grains. FA is an effective natural antioxidant as it possesses an aromatic phenolic core and an extended conjugated side chain, enabling it to scavenge free radicals and reduce oxidative damage, demonstrating considerable renoprotective effects in models of cisplatin-induced nephrotoxicity [8]. FA prevents ROS from damaging DNA and lipids. FA has been shown to act against many diseases through different pathways by regulating the signaling pathways of nuclear factor erythroid-2-related factor-2/heme oxygenase-1 (Nrf2/HO-1) and NF-κB, reducing oxidative damage and inhibiting inflammatory responses. FA possesses effective pharmacological impacts such as being an antioxidant, anti-cancer, anti-fibrotic, and anti-inflammatory agent [9, 10]. Moreover, it blocks the transforming growth factor beta (TGF-β)/small mothers opposing the signaling pathway for decapentaplegia (Smad), playing an antifibrotic role. It also has an anticancer activity by elevating p53 and decreasing Bax expression [11]. The therapeutic use of FA is hindered by inadequate aqueous solubility, fast metabolism, and restricted oral bioavailability, which limits its in vivo efficacy when given in its free form [8, 12]. Recently, studies have proven that using nano-encapsulated delivery technologies with ferulic acid makes it more efficient in the delivery and targeting of FA, increasing its oral bioavailability [13, 14].

Nanotechnology, which centers on the manipulation of matter at the nanoscale (1–100 nm), reveals distinctive features and applications. Various areas gain advantages, ranging from electronics to healthcare [15]. The integration of biological systems with nanomaterials is an emerging technique operating at the nanoscale, designed to optimize individual molecules and atoms within a specific dimension for various applications in health biotechnology, the industrial sector, agriculture, catalysis, and environmental research [16]. A limited size distribution of magnetic nanoparticles (MNPs) has emerged in recent years, garnering significant attention due to their applications in pharmaceutical delivery, bio-diagnostics, and also possessing extensive uses in electrochemistry, biomedicine, and biotechnology owing to their biocompatibility, natural abundance, and cost-effectiveness [17, 18]. They are easily modified to bind to biomolecules or contaminants preferentially. They have intrinsic biocompatibility and low toxicity, facilitating the successful utilization of iron oxide nanoparticles across various applications, in addition to their functionalization, which can augment stability and adsorption characteristics, hence substantially enhancing detection efficacy [19, 20]. The coating and functionality of MNPs with organic and inorganic coatings such as surfactants, polymers, silica, and carbon are used to enhance their biocompatibility not only by improving their quality and surface chemistry but also by their engagement with biological barriers, including the blood-brain barrier, cellular membranes, and vascular endothelium, which facilitates the nanoparticles’ transit through these barriers, as they posses extensive applications in biomolecular imaging, therapies, drug delivery, biomedicines, cancer treatment, cosmetic surgery, and molecular detection [21–23].

Carbon coatings have been extensively utilized owing to their superior thermal stability, biocompatibility, and chemical stability in ambient conditions, leading to good dispersibility and less aggregation than uncoated MNPs. Furthermore, several functional groups on the carbon shell surface can be readily modified and functionalized, enhancing their applicability under different conditions and improving their specific performance in varied applications [17, 24]. MNPs are regarded as very promising drug carriers, as therapeutic magnetite nanoparticles typically consist of three basic parts: a therapeutic load that performs a particular function in vivo; a coating on the iron oxide nanoparticle that promotes beneficial interactions within the body; and a magnetite core that serves as a carrier for therapeutic agents [25]. MNPs exhibit biocompatibility as iron oxides are naturally present in the human heart, spleen, and liver. This renders them non-toxic and biocompatible at physiological doses. They can carry the medications in active forms to locations where traditional pharmaceuticals cannot be accessed independently, thus reducing undesired side effects. Moreover, as they possess a high ratio of surface to volume, which allows for the optimisation of drug exposure at the target site with a minimal quantity, they mitigate potential toxicity concerns associated with the integrated medication [26]. Zhong et al. demonstrate that carbon-coated MNPs (Fe_3_O_4_-NPs@C) are biocompatible, safe, and decrease oxidative stress by acting as an antioxidant [27]. Recently, nanotechnology-based delivery systems have been progressively investigated to improve the stability, tissue targeting, and controlled release of nephroprotective medicines in cisplatin-induced acute kidney injury highlighting the potential of FA-loaded nanocarriers for renal protection. Magnetic Fe₃O₄ nanoparticles are promising options for drug carriers owing to their biocompatibility, extensive surface area, and capability for targeted administration, and have been effectively utilized to enhance anticancer drug delivery while minimizing systemic toxicity [28–31].

While cisplatin remains a nephrotoxic agent in cancer therapy, its progression of acute kidney injury (AKI) is primarily driven by oxidative stress and impaired targeted delivery, as evidenced by recent studies [1, 4], our study presents ferulic acid loaded with MNPs @C as a renoprotective and therapeutic agent in acute kidney injury caused by cisplatin by using FA as an antioxidant and MNP@C as a nanocarrier that enhances targeting drug delivery of ferulic acid in addition to its properties that decrease oxidative stress and improve red blood cell (RBC) status, which is strongly related to the treatment of AKI, no prior reports have combined FA loading with MNPs@C for synergistic renoprotection in cisplatin-induced AKI. Our study addresses this gap by demonstrating MNPs@C-enhanced FA delivery, improving targeting, mitigating oxidative damage, and restoring RBC status to advance AKI therapeutics.

## Materials and Methods

### Chemicals

Cisplatin, ferulic acid (purity > 99%), and sucrose were bought from Sigma Aldrich in the UK. Eprex (epoetin) was purchased from Janssen Pharmaceuticals. Ferric chloride (FeCl_3_.6H_2_O, 97%), ammonium hydroxide (NH_4_OH), ferrous chloride (FeCl_2_.4H_2_O, 99%), and ammonium acetate 25% (CH_3_COONH_4_) were obtained from Merck (Darmstadt, Germany).

### Synthesis and Functionalization of Fe_3_O_4_-NPs

Magnetic nanoparticles Fe_3_O_4_ were synthesized using the co-precipitation by utilizing FeCl_3_.6H_2_O and FeCl_2_.4H_2_O in a 3:1 molar ratio, then NH_4_OH (30%) was gradually added _4_ to the aqueous solution until the pH attained 10 at ambient temperature. After forming black iron oxide particles, Fe_3_O_4_, they were recovered from the liquid with a bar magnet and washed three times with 99% ethanol to provide the final particles. The resultant iron oxide Fe_3_O_4_ nanoparticles were subsequently dried at room temperature. These Fe_3_O_4_ particles were then coated with carbon hydrothermally by suspending Stoichiometric quantities of Fe_3_O_4_, sucrose, and CH_3_COONH_4_ (30%) mixed with 60 mL of distilled water. The resulting mixture was introduced into a 75 mL Teflon-sealed autoclave and subjected to a temperature of 190 °C for a duration of 4 to 12 h. The products were isolated from the solution with an external magnet and rinsed with deionized water and 99% ethanol many times. The black products were dried in a vacuum oven for 24 h at 60 °C [24]. According to Zhong et al., FA was loaded with some modifications: 100 mg of Fe_3_O_4_ was suspended in 10 ml of 99% ethanol (Merck, Germany) and combined with 70 ml of ethanolic FA (10 mg/ml). The resulting mixture was stirred for two hours using a mechanical stirrer (HG 15, Dihan, South Korea). Finally, the FA/Fe_3_O_4_-NPs@C was separated by centrifugation at 15,000 rpm (Hermle Z32 HK, Germany) and dried overnight at 45 °C [27].

### Characterization of Ferulic Acid-loaded Fe_3_O_4_-NPs@C

Fourier-transform infrared spectrophotometric analysis (FT-IR) of pure FA and Fe_3_O_4_-NPs@C was recorded and compared with spectra of FA-loaded Fe_3_O_4_-NPs@C by using a Shimadzu IR 435 spectrophotometer (Shimadzu Corporation, Kyoto, Japan) within the range of 400–4000 cm⁻¹ to study interactions between them. Transmission electron microscopy (TEM) analysis was carried out on a Talos F200i (Thermo-scientific) electron microscope utilizing an acceleration voltage of 200 kV to examine structural morphology and particle-size distributions using ImageJ software with Origin to analyze TEM images. An XRD pattern of the Fe_3_O_4_-NPs and Fe_3_O_4_-NPs@C has been performed and compared with FA/Fe_3_O_4_-NPs@C to study the crystalline structure and purity using the XPERT-PRO Powder Diffractometer system, which operates within a 2 theta range of 5° to 80°, featuring a minimum step size of 0.001° and a wavelength (Kα) of 1.54614 Å. The surface morphology was acquired by using a TESCAN MIRA field-emission scanning electron microscope (FE-SEM) at a landing energy of 5 kV and a current of 100 pA.

### Animals and Experimental Design

A total of 42 male Sprague-Dawley rats in filter-top cages weighed between 180 and 200 g and lived at 25 °C with humidity levels between 55% and 70%. They also had a cycle of lights and darks for 12 h. Before starting the study, the rats were administered a standard diet and complimentary access to water for one week to get used to their new environment.

Forty-two rats were subsequently allocated randomly into seven groups, with six rats per group. Group I (normal group): rats neither injected nor treated. Group Ⅱ (Positive control group of cisplatin): rats received a single intraperitoneal injection of cisplatin at a dosage of 6 mg/kg [32]. Group III (cisplatin and EPO): after 2 h of cisplatin induction at 6 mg/kg as a single dosage, rats received a single administration of 1000 IU/kg erythropoietin by intraperitoneal injection [33]. EPO was included as a positive nephroprotective control, as it attenuates cisplatin-induced acute kidney injury by reducing tubular apoptosis, oxidative stress, and inflammation. Additionally, it promotes tubular cell survival and regeneration, upregulates antioxidant enzymes (e.g., SOD, catalase), and provides erythropoiesis-independent tissue protection via EPO receptor activation in renal tubular cells. In this study, EPO is used to compare its renoprotective effect with that of the combination Group VI to evaluate possible synergistic effects. Furthermore, EPO was included to validate our novel FA-MNPs@C intervention (Group VII) against a clinically relevant standard on oxidative stress markers and RBC parameters in AKI [34–37].Group IV (cisplatin and FA): rats were given FA orally administered at a dosage of 50 mg/kg daily for 7 days, subsequent to cisplatin injection at a dosage of 6 mg/kg [38]. Group V (cisplatin and Fe_3_O_4_-NPs): Rats were administered a single intraperitoneal dosage of cisplatin at 6 mg/kg, followed by an intraperitoneal injection of Fe_3_O_4_-NPs at 5 mg/kg two hours post-injection [39]. Group VI (combination group): rats received an intraperitoneal injection of a single dose of 6 mg/kg of cisplatin. Two hours later, rats received a single dosage of 1000 IU/kg erythropoietin, 5 mg/kg Fe_3_O_4_-NPs, and daily oral administration of 50 mg/kg ferulic acid for 7 days [38]. GroupⅦ (cisplatin& FA/Fe_3_O_4_-NPs@C): rats were administered an intraperitoneal injection of cisplatin at a dosage of 6 mg/kg, followed by an intraperitoneal injection of FA/Fe_3_O_4_-NPs@C at a dosage of 5 mg/kg two hours post-injection. Following 7 days of cisplatin administration, the animals were subjected to a 12-hour fast with free access to water, after which they were euthanized.

### Preparation of Samples

Rats were weighed, subjected to an overdose of halothane, and subsequently euthanized at the end of the experiment. Before euthanasia, blood samples were drawn straight from the heart using a syringe and placed in plain collection tubes from each rat. These samples were used to measure the kidney function biomarkers serum creatinine and urea. The blood sample tubes were permitted to coagulate for 30 min, then centrifuged (JANETZKI T30, Germany) at 4000 rpm for 10 min to extract the serum, which is thereafter stored in a 2 ml Eppendorf tube and frozen at − 20 °C. Anticoagulant tubes containing ethylenediaminetetraacetic acid (EDTA) are also used to determine hematological parameters, including mean corpuscular volume (MCV), mean corpuscular hemoglobin (MCH), mean corpuscular hemoglobin concentration (MCHC), red blood cells (RBC), hemoglobin (Hb), and hematocrit (HCT). The kidneys of rats were removed and rinsed with saline. Each kidney was bisected using a lancet, and one half of the left kidney was preserved in 1 ml of RNAlater (Qiagen Inc., MD, USA) at − 80 °C for real-time PCR analysis. Evaluating gene expression for Bcl-2, Bax, Interleukin-6 (IL-6), GPX-4, and TGF-β. One half of the right kidney was promptly placed in a 10% neutral buffered formalin container to examine immunohistochemical (Bax) and histopathological analyses. The remaining right kidney was homogenized in a phosphate buffer (pH 7.4) to produce a kidney homogenate, which was stored at − 80 °C. For the assessment of oxidative stress and antioxidant levels, this homogenate was centrifuged at 4000 rpm for 15 min at 4 °C and then refrigerated at − 80 °C.

### Biochemical and Hematological Investigation

Renal function markers, including serum creatinine and urea, were measured using the Robonik Prietest Touch semi-automated biochemistry analyzer and kits from AGAPE Diagnostics (Switzerland). Hematological parameters Hb, RBC, HCT, MCV, MCH, and MCHC were measured, along with ferritin. Moreover, Bio-Diagnostics (Dokki, Giza, Egypt) kits for malondialdehyde (MDA) [40], superoxide dismutases (SOD) [41], catalase (CAT) [42], and reduced glutathione (GSH) [43] were used to colorimetrically measure their levels in renal tissue using the procedures outlined in the leaflets that came with the kits.

### Real Time-PCR

As directed by the manufacturer, total RNA was extracted from kidney tissue using the TRIzol reagent (Thermo Fisher Scientific, Inc., USA). Thereafter, total RNA was isolated and purified [44]. The Nanodrop spectrophotometer (München, Germany) was used to measure the amount of RNA. The Maxima first-strand cDNA synthesis kit (Thermo Scientific, USA) and random hexamer primers were used to convert two micrograms of total RNA into complementary DNA. Ten microliters of 2× SYBR Green master mix (Maxima, Thermo Fisher Scientific, USA), two microliters (150 ng) of template, two microliters (10 pmol) of each primer, and nuclease-free water made up the PCR reaction, which had a total volume of 20 µl. In the Arktik Thermal Cycler (USA), the samples were amplified using the following protocol: initial denaturation at 95 °C for 4 min. Followed by 40 cycles of denaturation (15 s) at 95 °C, annealing (30 s) at 55 °C, and extension (30 s) at 72 °C. As shown in Table 1, Vivantis (Selangor Dar Elhasan, Malaysia) created primers for the genes Bcl-2, IL-6, Bax, GPX-4, and TGF-β using NCBI resources. Each sample was analyzed in triplicate using the 2^−ΔΔCt^ technique as the cycle threshold (Ct) values of Bcl-2, Bax, IL-6, GPX-4, and TGF-β were normalized to GAPDH as an internal housekeeping control (ΔCt = Ct _target_ – Ct _GAPDH_). Relative mRNA expression was calculated by the 2 ^−ΔΔCt^ method, using the normal control group as the calibrator (ΔΔCt = ΔCt_sample_ – ΔCt _normal_) [32].

**Table 1:** Primer sequences for Real-Time PCR

Genes	Forward primer	Reverse primer	Accession number
BAX	5′-AAGAAGCTGAGCGACTGTGTCT-3′	5′-CAAAGATGGTCACTGTCTGC-3′	(NM_017059.2)
BCL2	5′-GTACCTGAACCGGCATCT-3′	5′-ATCAAACAGAGGTCGCA-3′	(NM_016993.2)
IL-6	5′-TCCTACCCCAACTTCCAATGCTC-3′	5′-TTGGATGGTCTTGGTCCTTAGCC-3′	(NM_012589.2)
TGF-β	5′-TGCTAATGGTGGACCGCAA-3′′	5′-CACTGCTTCCCGAATGTCTGA-3′	(NM_021578.2)
GPX-4	5′-GAGATGAGCTGGGGCCGTCTGA-3′	5′-ACGCAGCCGTTCTTATCAATGAGAA-3′	(NM_017165.4)
GAPDH	5′-TATCGGACGCCTGGTTAC-3′	5′-CTGTGCCGTTGAACTTGC-3′	(NM_017008.4)

### Histopathological Examination of Renal Sections

Immediately after being cut up, each rat’s kidney samples were placed in 10% buffered neutral formalin for a duration of 72 h to ensure complete penetration and preservation of renal architecture, preventing autolysis and shrinkage artifacts. Subsequently, the fixed tissues underwent dehydration using increasing concentrations of graded ethanol (70–100%), cleaned with xylene, and embedded in paraffin at 60 °C. Histological evaluation involved cutting sections of the kidney that were 5 μm thick using a rotary microtome and staining them with both hematoxylin and eosin to examine the renal tissues for standard assessment of tubular integrity, interstitial changes, and vascular congestion under the light microscope (low magnification ×100, scale bar 100 μm; high magnification ×400, scale bar 50 μm).

### Immunohistochemical Examination of Bax

Blocks of renal paraffin were divided into sections that were 4 μm thick. For antigen retrieval, the slides were deparaffinized with xylene, rehydrated with graduated ethanol concentration (100-95-75%), and then boiled in 10 mmol/l of citrate buffer (pH 6.0). The tissue slices were treated and incubated with primary monoclonal mouse antibodies targeting Bax (BioGenex, USA) following a 5-minute suppression of endogenous peroxidase activity with 3% H_2_O_2_. Following three PBS washes, the slides were treated with goat anti-rabbit IgG coupled with horseradish peroxidase for 60 min. They then reacted with 3,3′-Diaminobenzidine (DAB) chromogen and were counterstained with Mayer’s hematoxylin. As a negative control, the samples were treated and incubated with PBS rather than primary antibodies. The slides were examined at low magnification to determine the most consistently stained tissue sections because Bax antigen staining was limited to the nucleus [45].

### Statistical Analysis

The Statistical Package for the Social Sciences (SPSS) version 27 for Windows was used to do statistical analysis of the data, including the mean, standard deviation (SD), and p values. All data are presented as means ± standard deviation (SD). ANOVA and the Post Hoc LSD test were used to analyze the data statistically. The significance level for differences was set at *p* ≤ 0.05.

## Results

### Characterization of Ferulic Acid-loaded Fe_3_O_4_-NPs@C

TEM, FT-IR, and XRD were performed to characterize FA-loaded Fe_3_O_4_-NPs@C as follows:

#### TEM

**Fig. 1:**
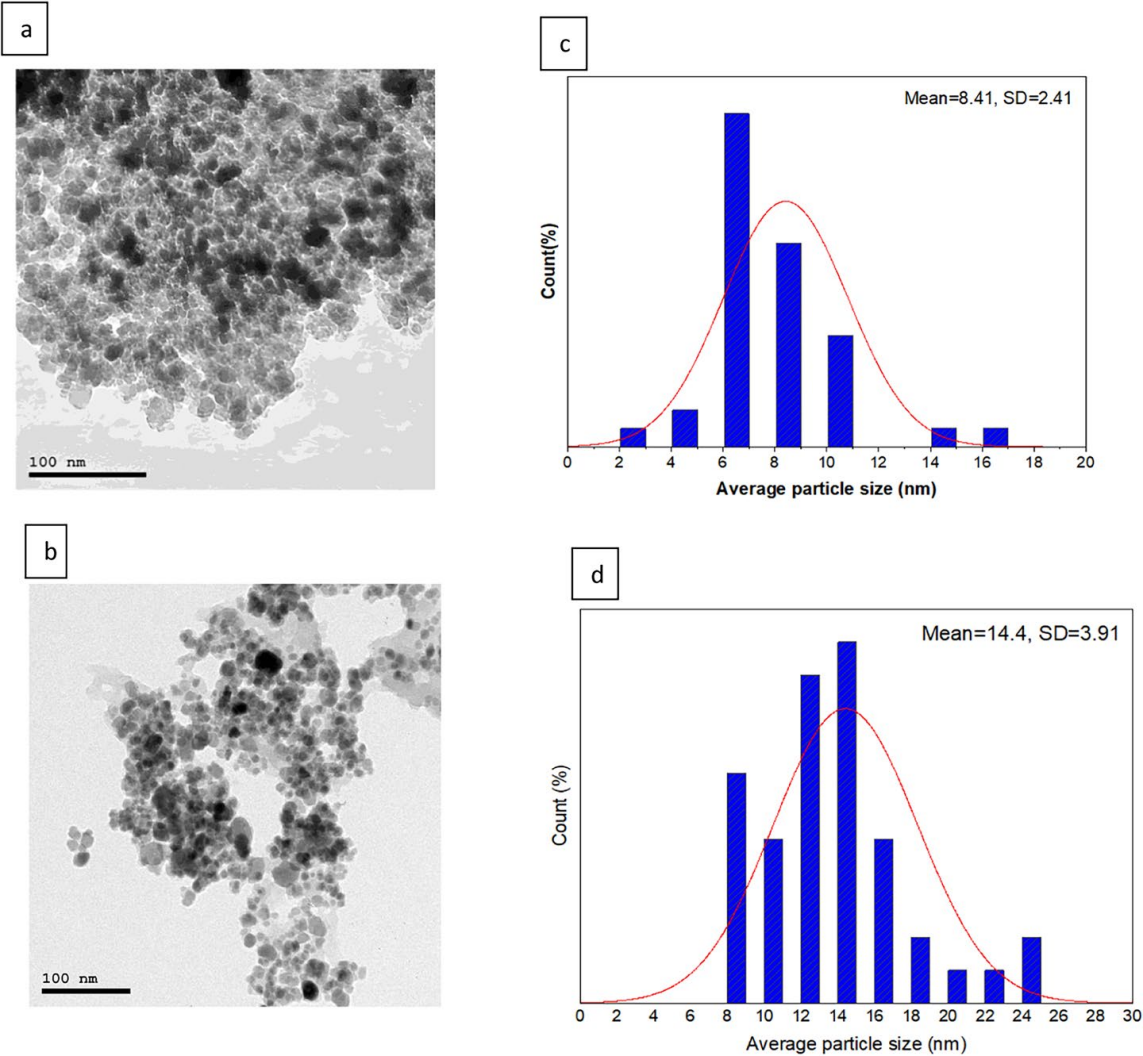
TEM images of magnetite nanoparticles functionalized with ferulic acid on their surface: (**a**) bare Fe3O4-NPs, respectively, (**b**) FA/Fe3O4-NPs@C; particle diameter distribution for (**c**) bare Fe3O4- NPs, respectively, (**d**) FA/Fe3O4-NPs@C

The morphology and size distribution of Fe_3_O_4_-NPs and FA-loaded Fe3O4-NPs@C were studied by TEM, as shown in Fig. 1. It can be seen that Fe_3_O_4_-NPs (a) have a spheroidal shape with a mean size of 6.057 ± 1.2 nm. Fe_3_O_4_-NPs also exhibit a high aggregation, which may be due to strong magnetic dipole-dipole interactions. After the coating functionalizes Fe_3_O_4_-NPs@C by ferulic acid (b), the particles showed a good monodispersity, less aggregated than Fe_3_O_4_-NPs, with an average size of about 7.83 ± 1.2 nm [24]. The coating appears in the grey carbon outer layer shell, and the black inner layer, a magnetite core. The electronic absorbance of the Fe_3_O_4_-NPs is higher than that of carbon; therefore, in the TEM results, Fe_3_O_4_-NPs seem darker than their carbon shell. The findings of Boustani et al. [46] supported our results.

#### FT-IR

**Fig. 2:**
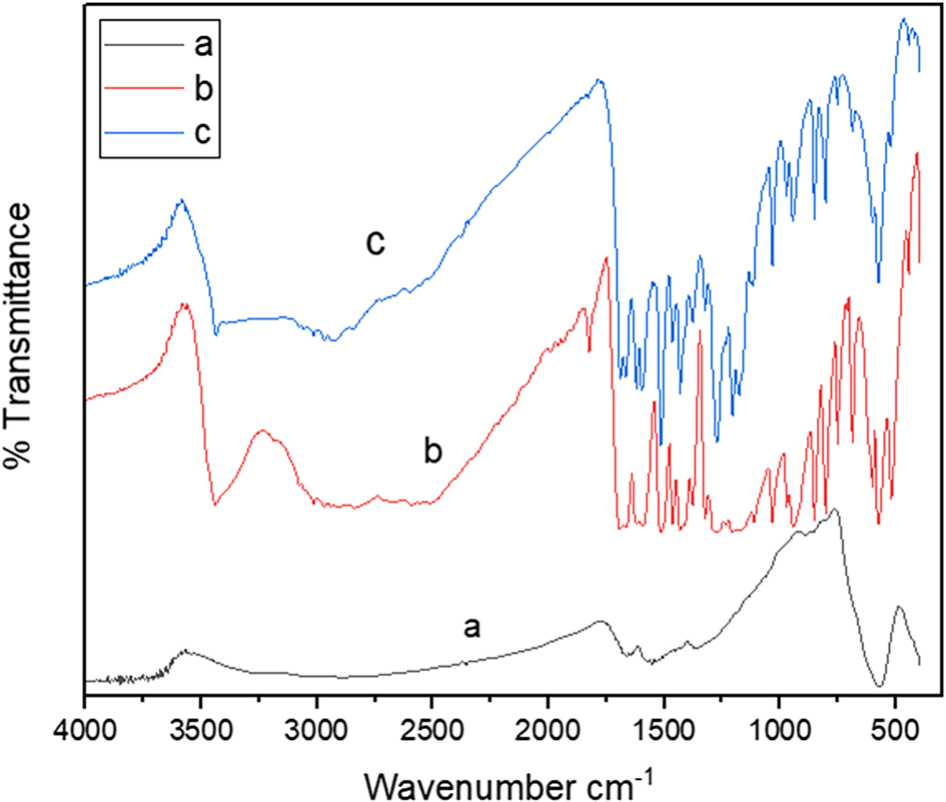
FTIR spectra of **(a)** hydrothermal carbon coating of Fe_3_O_4_-NPs, **(b)** Pure Ferulic acid, **(c)** FA/Fe_3_O_4_**-**NPs@C

 In Fig. 2, the FTIR spectra of Fe₃O₄@C (a) and pure ferulic acid (FA) (b) were obtained and subsequently compared with the spectra of FA-loaded Fe₃O₄@C (c) to investigate the molecular interactions within the nanoformulations. The spectra for FA-loaded Fe₃O₄@C (c) exhibited an overlay of the characteristic spectra of both FA and Fe₃O₄@C. In the FA-loaded Fe₃O₄@C, the peak observed at 3437.15 cm⁻¹ indicates O–H stretching [47], whereas the peaks at 1276.88, 1203.58,1516.05,1689.6/1666.5, and 1620.2. correspond to C–O, C–OH, C–C, C = O, and C = C stretching vibrations, respectively [48]. The previous specific peaks were absent in the spectra of the Fe₃O₄@C (a), suggesting that they might be attributed to ferulic acid included in the prepared nanoformulations. Based on the spectral analysis, the functional groups of FA on the surface of Fe₃O₄@C exhibited chemical characteristics nearly identical to those of pure FA. The study concluded that no chemical interactions occurred between the functional groups of ferulic acid and Fe₃O₄@C, as no molecular interactions observed could have changed the chemical structure of the FA.

#### XRD

**Fig. 3:**
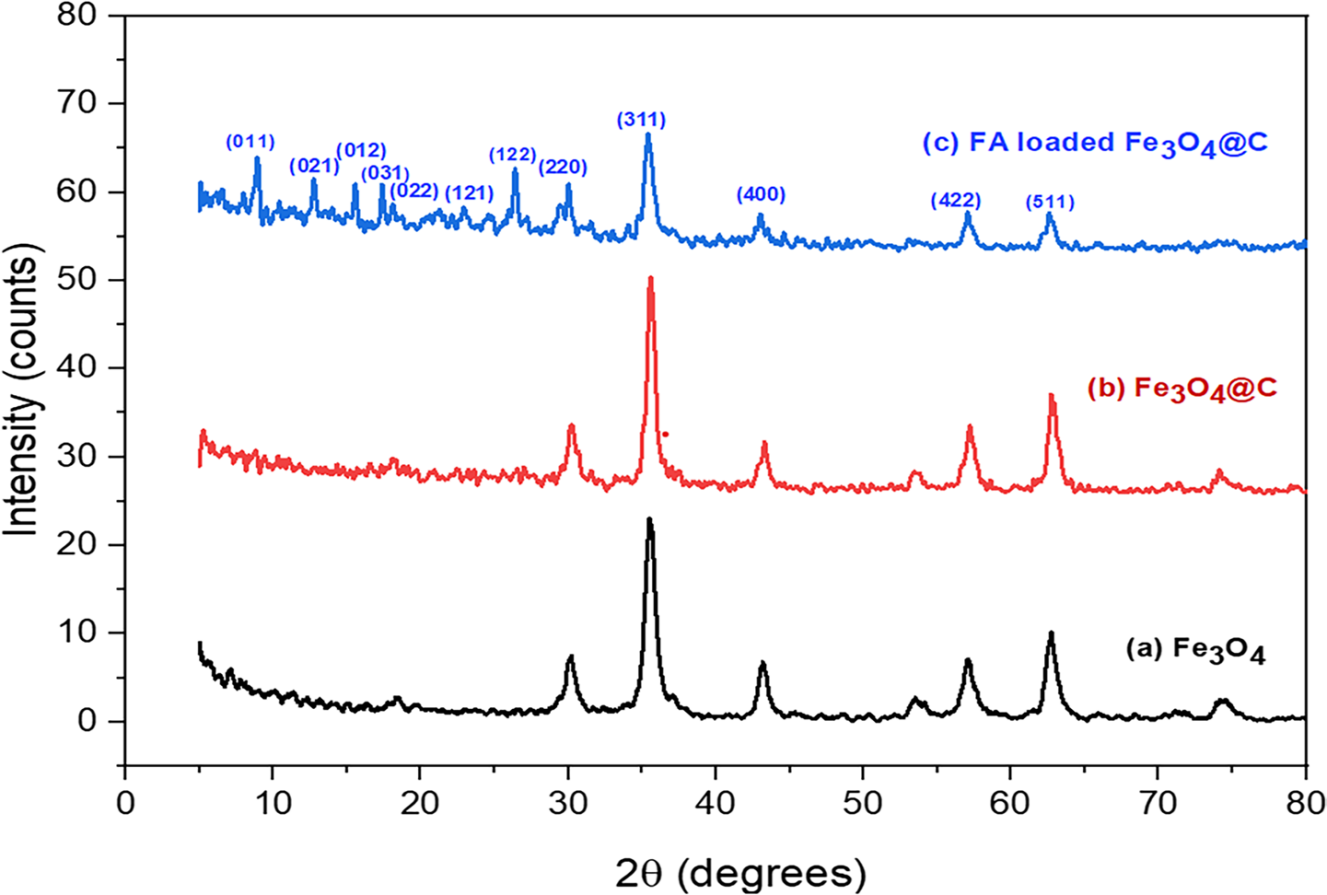
Comparison of XRD patterns of **(a)** bare Fe_3_O_4_**-**NPs, **(b)** hydrothermal carbon coating of Fe_3_O_4_-NPs, **(c)** FA/Fe_3_O_4_**-**NPs@C

The XRD patterns of Fe₃O₄ (a), Fe₃O₄@C (b), and FA-loaded Fe₃O₄@C (c) in Fig. 3 exhibited peaks at 2θ values of 30.2°, 35.49°, 43.2°, 57.2°, and 62.7°, which match the crystal planes of the Fe₃O₄ spinel structure. The peaks show that both Fe₃O₄ and Fe₃O₄@C keep the cubic spinel structure of magnetite even after being coated with carbon. This shows that the coating was done without changing the crystalline structure of the magnetite nanoparticles. The sharp peaks match the JSC 00–019-0629 card, which shows that the black magnetite is well-crystallized [49]. When ferulic acid (FA) was added to the carbon-coated magnetite, new peaks showed up at 2θ values of 9.01°, 12.83°, 15.63°, 17.31°, 18.07°, 23.01°, and 26.35°. These peaks matched those in the PDXL database (PDF Card No. 00–041-1606) [50]. These additional peaks show that FA has been successfully added to the nanoformulation and suggest that new crystalline phases or molecular ordering have formed. The XRD study shows that producing FA-loaded magnetite covered with carbon was effective, and both magnetite and FA kept their crystalline structure.

#### SEM

**Fig. 4:**
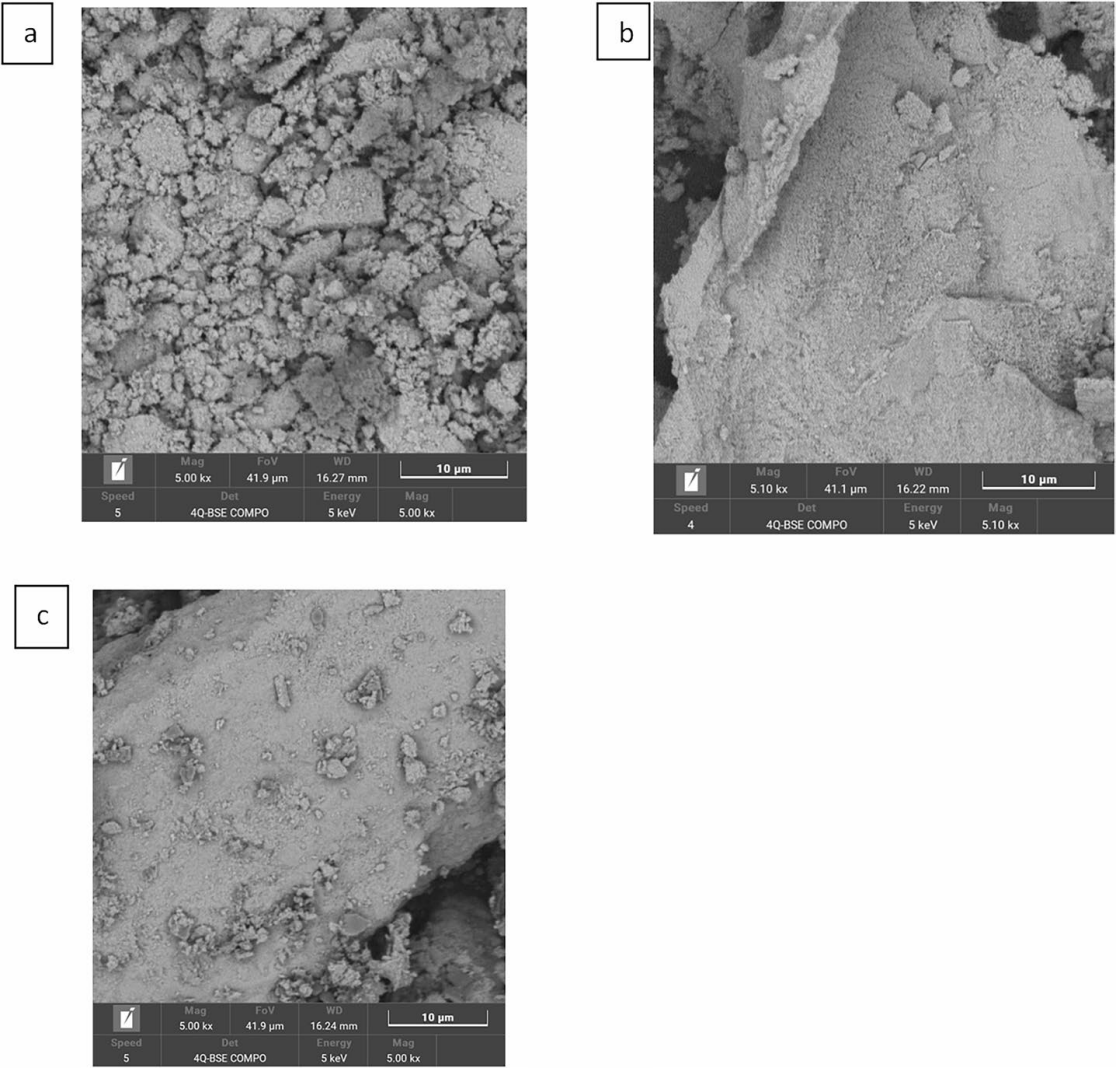
SEM of bare magnetite **(a)**, carbon-coated magnetite hydrothermally **(b)**, and ferulic acid-coated carbon-coated magnetite **(c)**, illustrating the surface characteristics and structural differences between the three forms

SEM images **(**Fig. 4**)** further illustrate the effect of coating and loading. Bare Fe₃O₄-NPs **(a)** show a rough surface with clearly visible pores, whereas **(b)** Fe₃O₄@C exhibits a smoother surface and partial pore coverage. **(c)** FA-loaded Fe₃O₄@C displays additional smoothing and reduced visible pore openings, consistent with deposition of an organic FA-containing layer. Quantitative SEM–ImageJ analysis of surface and pore regions (Supplementary Table S1) shows a substantial decrease in pore %Area and brightness from bare to coated and FA-loaded states, confirming progressive surface coating and material loading. These results aligned with previous studies of study morphology of the surface of magnetite Nps [51–53].

### Effectiveness of EPO, FA, Fe₃O₄ Nanoparticles, EPO/FA/Fe₃O₄ NPs Combination and FA-loaded Fe₃O₄@C on Biochemical Parameters of Cisplatin-induced Nephropathy

The therapeutic effects of the EPO, FA, Fe₃O₄, EPO/FA/Fe₃O₄ nanoparticles combination, and FA-loaded Fe₃O₄@C on cisplatin-induced renal impairment were evaluated by evaluating serum creatinine and urea concentrations, as detailed in Table 2. Serum creatinine and urea levels were significantly higher in the cisplatin group than in the normal, healthy group. In contrast, all treated groups showed a significant reduction in these renal biomarkers (*p* ≤ 0.05) in comparison to the cisplatin group after 7 days of treatment. Among the treatments, the EPO/FA/Fe₃O₄ nanoparticles combination and FA-loaded Fe₃O₄@C demonstrated the most effective reduction in creatinine and urea levels, restoring renal functions. Still, the urea value in the final treatment group remains approximately 10 mg/dL higher than the Normal group; this slight difference lies within a range that is unlikely to have a meaningful impact on overall renal function in vivo, but this indicates partial rather than complete normalization.

**Table 2:** FA/Fe_3_O_4_-NPs@C and other treatments affect renal function indicators of rats IP administrated with cisplatin

Variables (Mean ± SD)	Normal	Cisplatin	Cis + EPO	Cis + FA	Cis + Fe_3_O_4_-NPs	Cis + Compination	Cis + FA/Fe_3_O_4_-NPs @C
Creat (mg/dl)	0.36 ± 0.07^#^	0.93 ± 0.74^*^	0.51 ± 0.03^*#^	0.46 ± 0.03^*#^	0.39 ± 0.038^#^	0.37 ± 0.01^#^	0.36 ± 0.02^#^
Urea(mg/dl)	22.1 ± 0.91^#^	116.88 ± 39.27^*^	65.96 ± 3.14^*#^	34.1 ± 8.13^#^	32.83 ± 4.81^#^	31.22 ± 3.09^#^	28.71 ± 4.3^#^

Each value is expressed as mean ± standard deviation, *N* = 6

#: Markedly significant compared to cisplatin *p* ≤ 0.05

*: Markedly significant in comparison to normal *p* ≤ 0.05

### Effectiveness of EPO/FA/Fe₃O₄ Nanoparticles Combination and FA-loaded Fe₃O₄@C on Hematological Parameters of Cisplatin-induced Nephropathy

The efficacy of the treated groups in reducing anemia brought on by acute kidney injury from cisplatin was assessed and is shown in Table 3. Ferritin, RBC count, hematocrit, RDW, MCV, MCH, MCHC, and hemoglobin levels were significantly lower in the cisplatin group than in the normal group (*p* ≤ 0.05). On the other hand, ferritin, hemoglobin, hematocrit, RBC count, and RDW improved moderately significantly after ferulic acid treatment. Ferritin and hematological parameters showed notable improvements in the iron oxide nanoparticles groups, which included magnetite, combination therapy, and FA-loaded Fe₃O₄@C. FA-loaded Fe3O₄-NPs@C showed the greatest therapeutic effiefficiency.

**Table 3:** FA/Fe_3_O_4_-NPs@C and other treatments affect ferritin and related hematological parameters of rats IP injected with cisplatin

Variables (Mean ± SD)	Normal	Cisplatin	Cis + EPO	Cis + FA	Cis + Fe_3_O_4_-NPs	Cis + compination	Cis + FA/Fe_3_O_4_-NPs @C
Ferrtin (ng/ml)	193.17 ± 1.67^#^	112.78 ± 2.98^*^	185.2 ± 3.14^*#^	198.6 ± 8.64^#^	204.88 ± 1.2^*#^	206.83 ± 2.54^*#^	253.78 ± 9.05^*#^
Hb (g/dl)	14.41 ± 0.31^#^	8.53 ± 0.79	13.64 ± 1.06^#^	13.78 ± 0.2^#^	14.08 ± 1.57^#^	14.18 ± 0.52^#^	14.41 ± 0.34^#^
hematocrit%	47.18 ± 1.59^#^	38.3 ± 4.48^*^	45.09 ± 1.87^#^	46.31 ± 2.03^#^	46.69 ± 2.17^#^	46.93 ± 2.26^#^	47.04 ± 1.2^#^
RBCS count (millions/ul)	6.71 ± 0.07	5.8 ± 0.2^*^	6.58 ± 0.27	7.25 ± 0.85^#^	7.57 ± 1.47^#^	7.61 ± 1.47^#^	7.97 ± 1.64^*#^
MCV (fl.)	69.71 ± 2.62^#^	58.76 ± 9.37^*^	61.29 ± 8.25	64.09 ± 7.83	64.13 ± 7.41	65.57 ± 8.02	66.85 ± 8.89
MCH (pg)	21.03 ± 0.84^#^	15.69 ± 2.08^*^	17.28 ± 2.73^*^	17.34 ± 2.53^*^	18.75 ± 2.1^#^	19.87 ± 3.07^#^	20.44 ± 1.06^#^
MCHC (g%)	30.08 ± 0.4^#^	26.73 ± 0.73^*^	27.99 ± 1.54^*^	28.05 ± 2.2^*^	29.12 ± 1.36^#^	29.76 ± 1.19^#^	30.17 ± 2.35^#^
RDW (%)	14.32 ± 1.51^#^	21.17 ± 3.27^*^	16.81 ± 1.91^*#^	16.54 ± 2.67^#^	15.85 ± 1.5^#^	15.39 ± 1.14^#^	13.88 ± 1.36^#^

Each value is expressed as mean ± standard deviation, *N* = 6

#: Markedly significant compared to cisplatin *p* ≤ 0.05

*: Markedly significant in comparison to normal *p* ≤ 0.05

### Impact of Various Therapies on Antioxidant and Renal Oxidative Stress

**Fig. 5:**
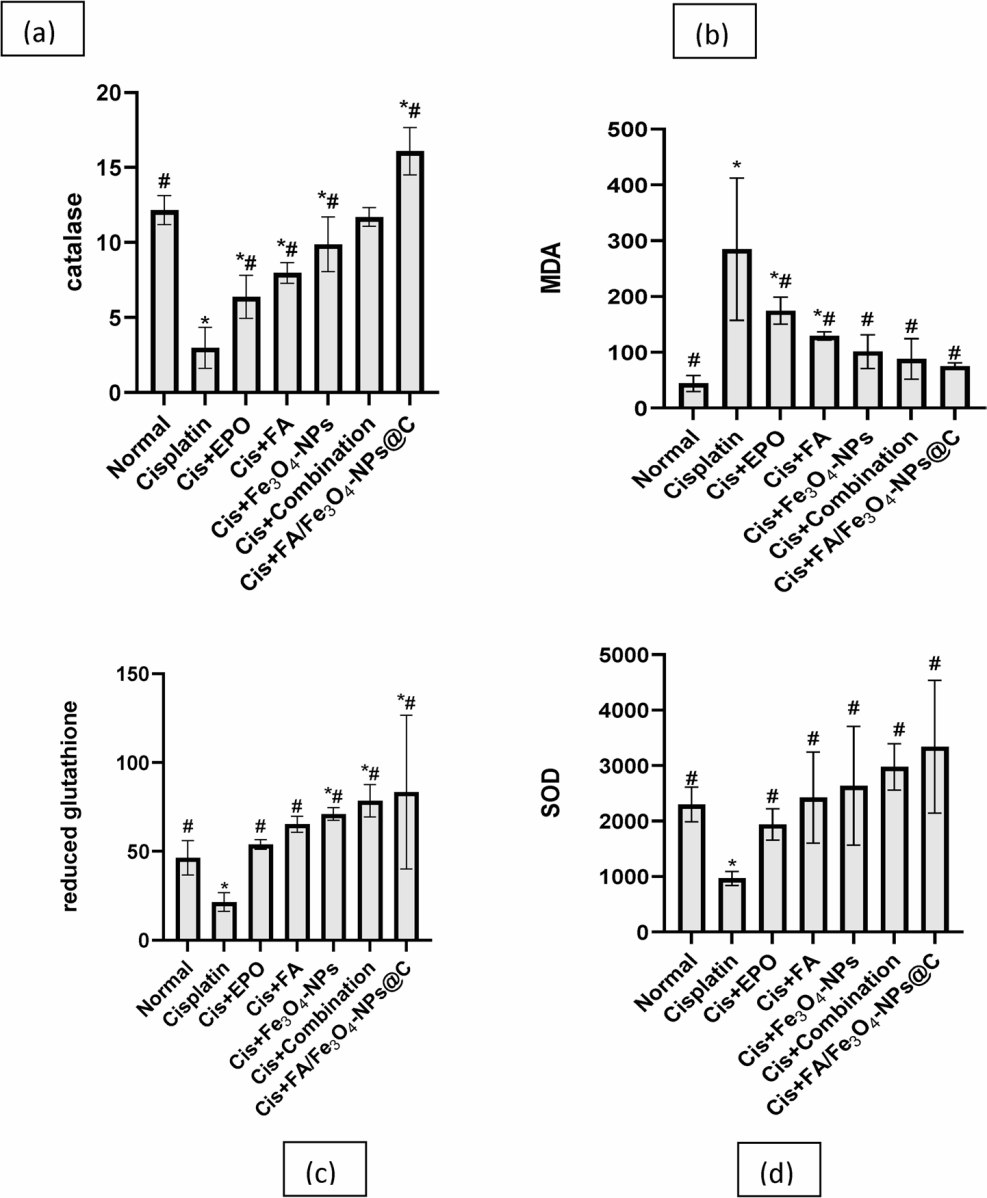
Comparison between treated groups against cisplatin and control normal group based on renal oxidative stress and antioxidant indicators as follows: (**a**) CAT (U/g tissue), (**b**) MDA (nmol/g tissue), (**c**) GSH (mg/g tissue), and (**d**) SOD (U/g tissue) #: Markedly significant compared to normal at *p* ≤ 0.05 ⁎: Markedly significant in comparison to cisplatin at *p* ≤ 0.05

Figure 5 illustrates the assessment of renal concentrations of MDA, SOD, CAT, and GSH to evaluate the therapeutic efficacy of the therapies, which include EPO, FA, Fe₃O₄ nanoparticles, the EPO/FA/Fe₃O₄ combination, and FA-loaded magnetite coated with carbon. The use of cisplatin injection yielded a markedly substantial elevation in MDA and reduction in SOD, CAT, and GSH relative to both the normal and the treated groups. All treated groups exhibited a significant decrease in MDA levels (*p* ≤ 0.05), whereas SOD, CAT, and GSH levels were considerably increased (*p* ≤ 0.05) in comparison to the cisplatin group. Among these treatments, FA-loaded Fe₃O₄@C had the most significant therapeutic efficacy.

### Effect of Different Treatments on Bcl-2, IL-6, Bax, GPX-4, and TGF-β Gene Expression

**Fig. 6:**
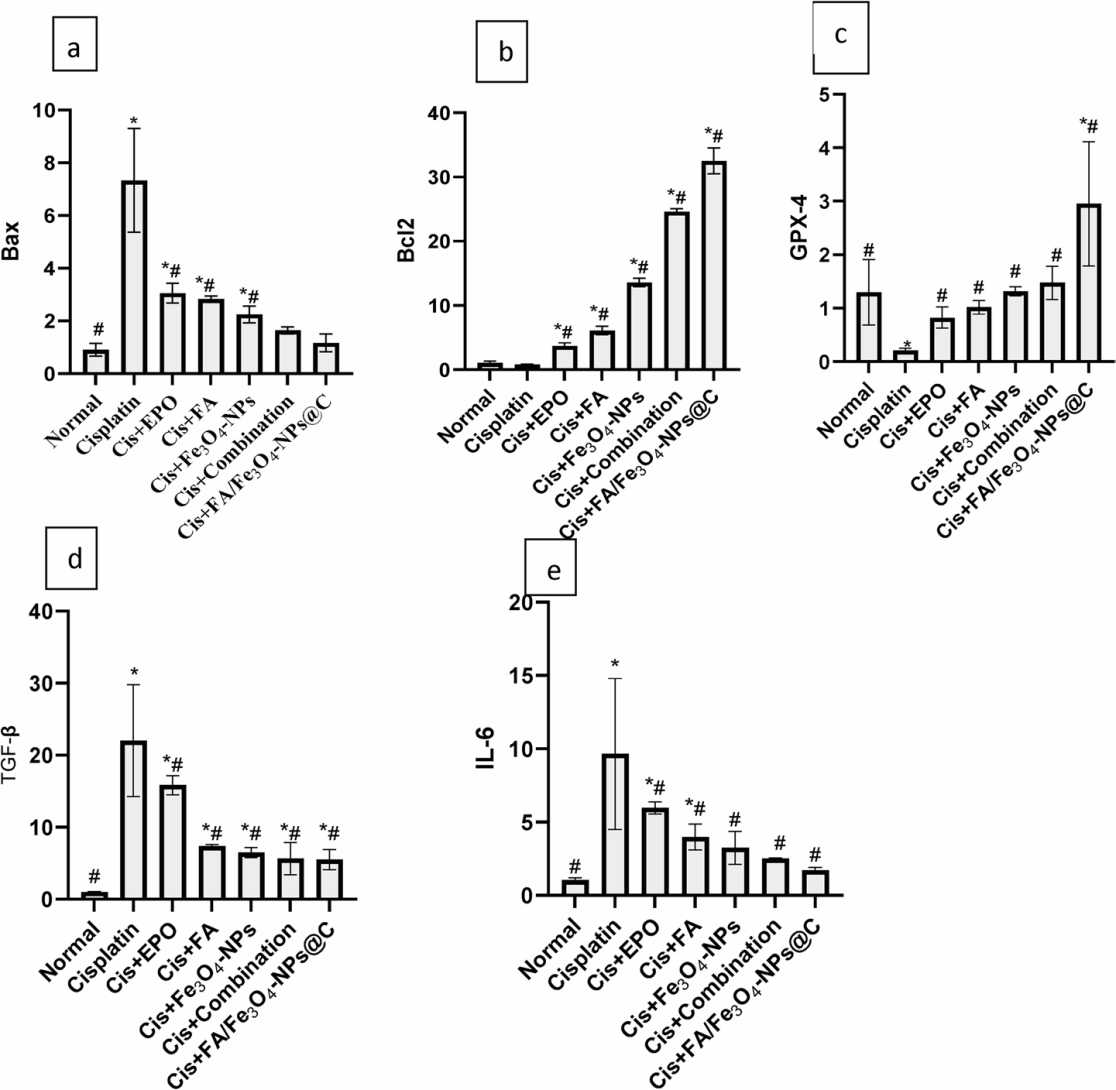
Effects of treatment with FA/Fe_3_O_4_**-**NPs@C and other drugs on the amounts of mRNA of **(a)** Bax, **(b)** Bcl-2, **(c)** GPX-4, **(d)** TGF-β, and **(e)** IL-6 in the kidneys of rats administered cisplatin with normalization to GAPDH as the housekeeping gene #: Markedly significant in comparison to cisplatin at *p* ≤ 0.05 *: Markedly significant compared to the control normal at *p* ≤ 0.05

The injection of cisplatin downregulated the expression of Bcl-2 and GPX-4 while increasing the Bax, IL-6, and TGF-β expression in kidney tissue compared to normal healthy rats. The effects of cisplatin were attenuated by treatment with EPO, FA, Fe_3_O_4_-NPs, the combination, and FA-loaded Fe_3_O_4_-NPs coated with carbon, as shown in Figs. 6. The cycle threshold (Ct) values of Bcl-2, Bax, IL-6, GPX-4, and TGF-β (Fig. 6a–e) were normalized using GAPDH as an internal housekeeping control by the 2^-ΔΔCT^ technique. In the cisplatin group, pro-apoptotic/pro-inflammatory genes (Bax, IL-6, TGF-β) increased markedly, while anti-apoptotic/antioxidant genes (Bcl-2, GPX-4) decreased. This pattern matches published cisplatin-induced AKI profiles [3–55]. Treatment, particularly with FA-loaded Fe₃O₄ NPs@C, significantly reversed these changes toward control levels (*p* < 0.05). This finding aligns with Bax immunohistochemistry and oxidative stress markers (GSH, SOD, catalase, MDA). The gene expression results are thus validated at the transcript, protein, and functional levels.

### Histopathological and Immunohistochemical Observations

#### Kidney Histopathological Observations

**Fig. 7:**
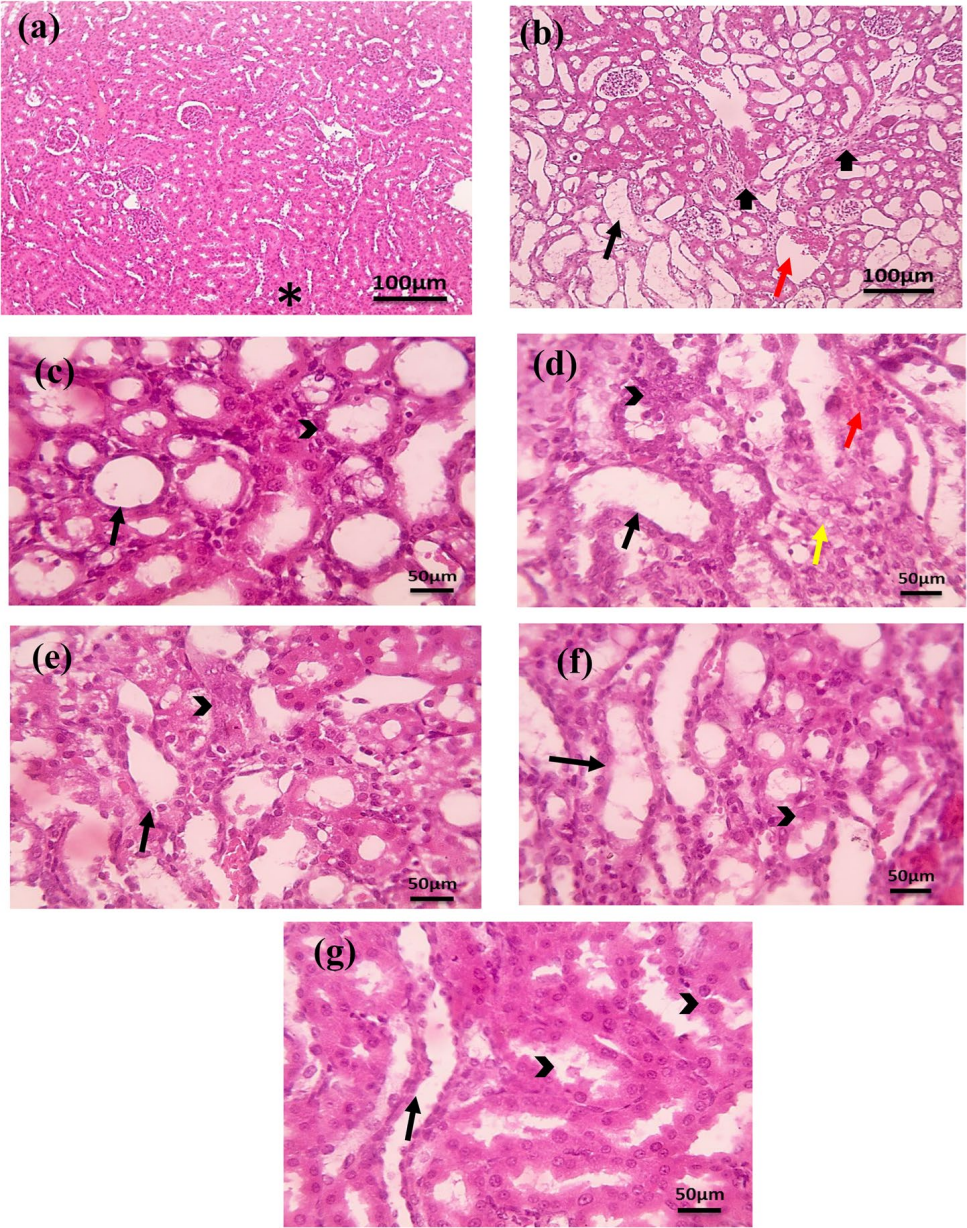
Microscopic images of H&E-stained kidney sections of **(a) **the control normal group. **(b)** cisplatin-injected group. **(c)** EPO Treated group** (d) **FA-treated group. **(e) **Fe_3_O_4_-NPs treated group. **(f)** combination group. **(g) **FA/Fe_3_O_4_-NPs@C treated group. Low magnification: 100X, bar 100; high magnification: 400X, bar 50

As shown in Fig. 7, the histopathological analysis revealed that the control group exhibited normal renal histology with no cortical and corticomedullary junction lesions (*). In contrast, the cisplatin group showed severe renal injury characterized by diffuse tubular dilation (thin black arrows), atrophy, congested blood vessels (red arrows), interstitial fibrosis (thick black arrows) in the cortex and corticomedullary junction& medulla, and mild signs of tubular regeneration (arrowheads) characterized by epithelial cells with more basophilic nuclei forming solid nodules. Treatment effects varied across groups: EPO treatment group led to significant tubular dilation and atrophy (thin black arrows) with mild regeneration (arrowheads); the FA treatment group showed a slight decrease in tubular dilation (thin black arrows) in the corticomedullary junction, exhibited mild degeneration (yellow arrows) and showed signs of regeneration (arrowheads) characterized by epithelial cells with large more basophilic nuclei alongside congested vessels (red arrows); Fe_3_O_4_-NPs treatment group showed moderate tubular dilation (thin black arrows) with mild signs of tubular regeneration (arrowheads) characterized by epithelial cells with large more basophilic nuclei. FA-Loaded Fe₃O₄-NPs@C treatment group displayed mild tubular dilation (thin black arrows), degeneration (arrowheads). Meanwhile, the EPO/FA/Fe₃O₄-NPs combination treatment resulted in moderate tubular dilation (thin black arrows) with a regenerative response (arrowheads) in the epithelial cells characterized by epithelial cells with large, more basophilic nuclei. Low magnification X: 100 bar 100 and high magnification X: 400 bar 50.

#### Immunohistochemical Staining of Bax

**Fig. 8:**
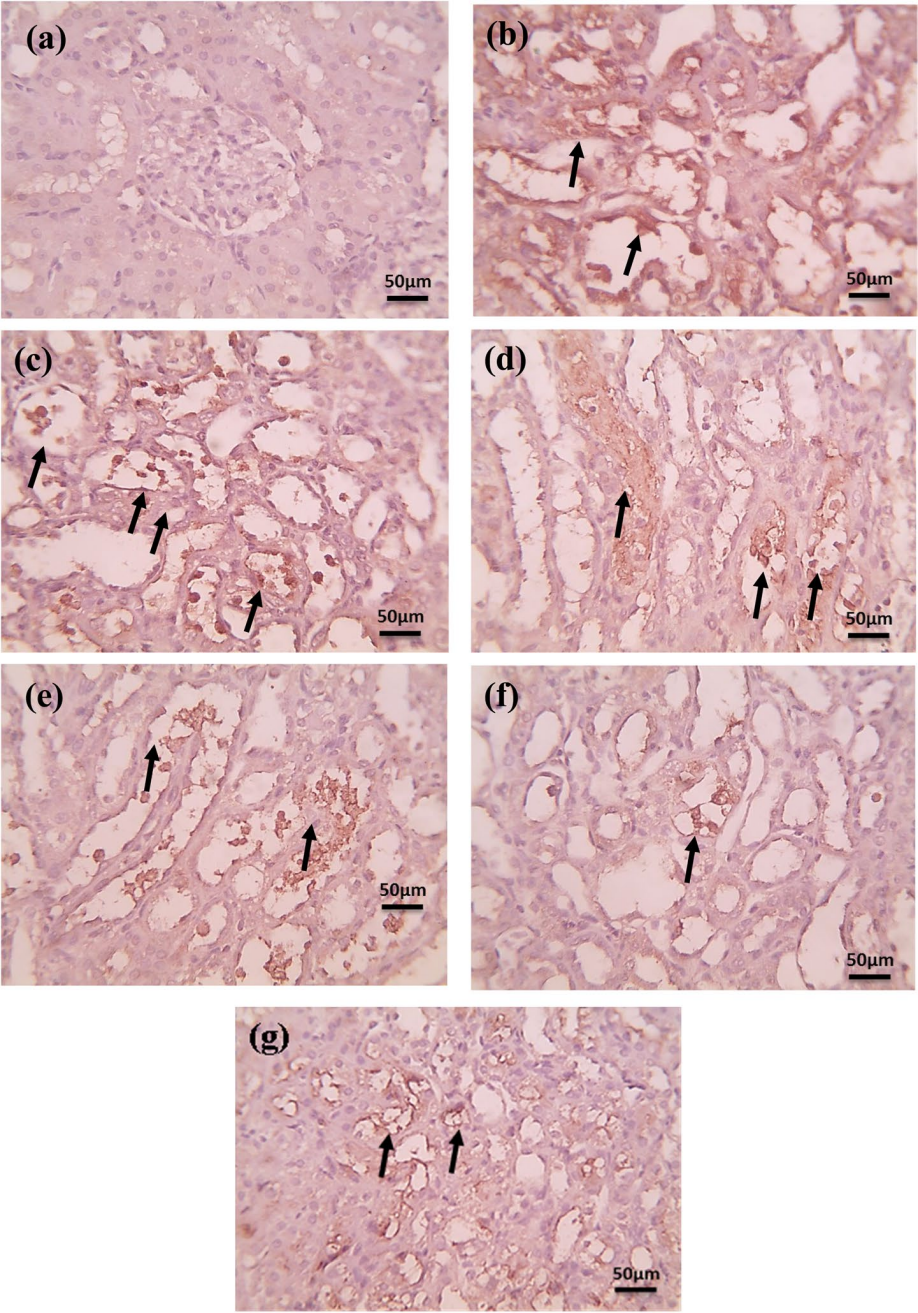
Microscopic images of immunostained kidney sections for BAX (brown) reveal negative tubular expression in (**a**) the control normal group. Strong positive brown tubular expression in the (**b**) cisplatin group. Slightly decreased positive brown tubular expression in treated groups with (**c**) EPO and (**d**) FA. Moderate positive brown tubular expression in the treated group with (**e**) Fe_3_O_4_-NPs. Mild positive brown tubular expression appears in treated groups with (**f**) FA/Fe_3_O_4_-NPs@C. Slightly higher positive brown tubular expression appears in the combination group (**g**). Black arrows point to positive staining. IHC counterstained using Mayer’s hematoxylin. High magnification: 400X, bar 50

Microscopic pictures of immunostained renal sections against BAX in Fig. 8 showed negative tubular expression in the normal group, strong positive brown tubular expression in the cisplatin group, and slightly decreased positive brown tubular expression in the treated groups (FA & EPO). The Fe_3_O_4_-NPs-treated group showed moderate positive brown tubular expression. Mild positive brown tubular expression appeared in treated groups with FA-Loaded Fe_3_O_4_-NPs@C. Slightly higher positive brown tubular expression appeared in the treated group with EPO/FA/Fe₃O₄-NPs combination (Low magnification 100X: bar 100 and high magnification: 400X bar 50).

## Discussion

Magnetic nanoparticles (MNPs) serve as carriers in drug delivery due to their numerous advantages, making them a very promising drug carriers. They are biocompatible, as iron oxides, specifically magnetite, naturally occur in the human heart, spleen, and liver, rendering them non-toxic and biocompatible [26]. The coating establishes a barrier that inhibits the aggregation of nanoparticles and facilitates their dispersion in physiochemical fluids [22]. Also considered a means to protect and stabilize the magnetic nanoparticles (MNPs) [26]. Carbon is considered to be an exceptional material due to its biocompatibility, stability across a wide pH range, and ease of surface modification make carbon a great material. Carbon-coated magnetite nanoparticles are stable in alkaline and acidic conditions, biocompatible, nontoxic, and saturation magnetized [24]. The functionalization of MNPS gives NPS enhanced stability in physiological environments, stealth characteristics, and vector targeting features [22]. Ferulic acid, despite its potent antioxidant and anti-inflammatory properties, suffers from poor aqueous solubility, rapid metabolism, and low bioavailability, which significantly limit its therapeutic efficiency in vivo when administered in its free form [56]. Therefore, functionalization with ferulic acid, which is loaded onto magnetic nanoparticles Fe_3_O_4_-NPs@C as a nanocarrier, enhances solubility, prolongs half-life, improves pharmacokinetic properties, and reduces toxicity. MNPs possess a large surface area, enabling the delivery of drugs in active forms to sites inaccessible to conventional medications, thereby minimizing undesirable side effects through smaller drug quantities and maximizing exposure at the target location. Consequently, any toxicity concerns associated with the integrated drugs can be avoided [25, 26]. At times, the preferred strategy involves unspecific physical sorption, which causes less stable conjugation to promote the disintegration of the nano-system [21], and this is what was achieved in the synthesis of FA-loaded Fe_3_O_4_-NPs@C and confirmed by IR in our study as magnetic nanoparticles (MNPs) must exhibit compatibility and biodegradability within the body for therapeutic purposes along with its metabolism as iron ions are sourced from the body’s iron storage and subsequently integrated into erythrocytes as components of hemoglobin within these particles [22]. MNPs were prepared using the co-precipitation method and coated with carbon using the hydrothermal method, and then FA-loaded Fe_3_O_4_-NPs@C according to Zhong et al. [27].

This study evaluated EPO, FA, Fe_3_O_4_-NPs, their combination, and FA/Fe_3_O_4_-NPs@C treatment groups alongside the control group to assess their efficacy in mitigating AKI induced by cisplatin nephrotoxicity. Cisplatin is mainly cleared by the kidneys, accumulating in the kidneys rather than in other organs. Despite its chemotherapeutic effect in cancer treatment, renal-accumulated cisplatin causes nephrotoxicity by intracellular stresses, including oxidative stress, DNA damage, and mitochondrial impairment [32]. Also, Ruixue et al. [39] reported that anemia developed in AKI, which causes insufficient EPO production, a point that has limited attention and decreased iron reabsorption, which is responsible for erythropoiesis. The earlier research indicated that cisplatin was intraperitoneally administered to rats at a dose of 6 mg/kg, which in our study induced a significant elevation in serum creatinine and urea levels with a substantial decrease in hemoglobin and RBC count after 7 days of administration. Also, the renal toxicity of cisplatin was confirmed in the histopathological alterations in our findings, comparable findings were reported earlier [57]. The accumulation of ROS, the build-up of lipid peroxidation products in the kidney, and the inhibition of the antioxidant system are believed to be the main mechanisms of cisplatin nephrotoxicity. In our study, the treated groups attenuated these changes caused by cisplatin-induced nephrotoxicity, which was confirmed by a significant decrease in urea and creatinine levels. Also, there was an elevation in Hb, ferritin, and RBC count after 7 days of treatment. Moreover, there was an improvement in histopathological findings in these treated groups, which aligned with the previous histopathological findings [32]. The treatments targeted pathways associated with oxidative stress and inflammation with the iron supplementation, as supported by previous studies [9, 39, 58, 59].

The main mechanisms of cisplatin-induced acute renal injury are thought to be the production of reactive oxygen species, the accumulation of lipid peroxidation products in the kidneys, and the suppression of antioxidant systems. Cisplatin is converted within the cell into a highly reactive form that engages with thiol-containing antioxidant molecules, including glutathione. Consequently, glutathione depletion leads to increased oxidative stress in the cells. Cisplatin may cause mitochondrial dysfunction through an impaired respiratory chain and reduced antioxidant defense mechanisms, including GSH, SOD, and CAT [60], which explained our results, which showed that the effects of cisplatin on the 7th day after injection increased renal MDA levels and decreased renal SOD, CAT, and GSH levels. This effect was nearly completely mitigated by the combination of cisplatin and FA-Loaded Fe₃O₄@C, which improves anti-inflammatory, lowers oxidative markers, and restores antioxidant defenses like SOD, GSH, and CAT when compared to their induction separately. The data from our investigation indicated the synergistic effect of each active ingredient in the combination therapy group, whereby EPO mitigated AKI by suppressing oxidative stress. EPO is safeguarded not only against renal nephrotoxicity and histological and biochemical alterations but also against the deterioration of renal function, corroborating other research that indicated EPO diminishes oxidative stress resulting from the infiltration of inflammatory cells into these organs. EPO diminishes the activation of the NF-kB pathway, which is stimulated by ROS and proinflammatory cytokines, as evidenced by our study’s observation of reduced proinflammatory IL-6 levels relative to the cisplatin group. Additionally, EPO preserves cell membrane integrity against oxidative stress, enhances antioxidant enzyme activities, and lowers MDA and free radical levels. These findings were previously reported for EPO [58, 61]. According to our study, by reestablishing redox balance and enhancing mitochondrial dynamics, FA mitigates renal impairment, which is supported by increased activity of antioxidant defense system enzymes like SOD and CAT. According to findings by Li et al. [59], Ferulic acid’s phenolic nucleus and long conjugated side chain allow it to scavenge free radicals, shielding DNA and lipids from oxidation driven by ROS produced during tissue injury. Consequently, in our study, this protection significantly inhibited lipid peroxidation and reduced malondialdehyde levels disrupted by cisplatin. Prior research has demonstrated that ferulic acid has therapeutic effects for inflammatory conditions, given the correlation between oxidative damage and inflammation [9] so the FA was introduced as a potent direct scavenger of free radicals [62–65].

Cisplatin may stimulate NF-kB by generating ROS and encouraging the nuclear translocation of Nrf2 [32]. In the cisplatin group, inflammatory mediators such as IL-6 have a significant expression, which validates the activation of the Janus kinase signal transducer and activator of transcription (JAK/STAT) molecular pathway and causes inflammation [66, 67]. FA is an anti-inflammatory agent that inhibited the NF-kB pathway [68, 69]. Our findings confirmed a significant decrease in expression of IL-6 in FA, combination, and FA-loaded Fe_3_O_4_-NPs@C. Transcription of IL-6 is associated with the activation of NF-kB. The suppression of this mechanism reduces the inflammatory mediator IL-6. Magnetite also diminishes renal function, as iron supplementation post-anti-inflammatory treatment is crucial for the recovery of acute kidney injury (AKI), as suggesting by our study that magnetite promoted erythrocyte production and enhanced hemoglobin levels, which indicated AKI recovery, suggesting the upregulation of EPO by the kidneys, which is subsequently transported to the bone marrow via the inhibition of this pathway, which decreases the inflammatory mediator IL-6.

Fibrosis reflects the process of wound healing. When injuries occur, fibrosis develops into a maladaptive healing mechanism in disease models, including acute kidney injury. The shift from pericytes to myofibroblasts is fundamental to the disease process [70]. Extracellular matrix (ECM) components are formed more easily when inflammatory mediators, such as transforming growth factor-β1 (TGF-β1), stimulate angiogenesis and myofibroblasts [71, 72]. Research has shown that TGF-β1 aggravates renal damage following cisplatin-induced injury. In our results, TGF-β1 expression was markedly elevated in the renal tissues of the cisplatin group on the 7th day of dosing, corroborating the findings of Lara et al. [73], who found that cisplatin elevates TGF-β1 levels, which promotes the production of extracellular matrix proteins and their deposition, resulting in glomerulosclerosis, tubular atrophy, and interstitial fibrosis. Both Smad-dependent and independent pathways are initiated by TGF-β, which causes various biological reactions. TGF-βRI is activated when TGF-β1 binds to its receptor, TGF-β receptor type II (TβRII). This creates a heterodimer that makes it easier for Smad2 and Smad3 to phosphorylate and then attach to Smad4. Then, Smad2 and Smad3 are carried to the nucleus, where they cause renal fibrosis [74]. Our findings confirmed renal fibrosis by histologically examining the cisplatin group. According to our data, EPO has shown a significant decrease in TGF-b1 expression, indicating its anti-fibrotic activity. These findings align with those of Kwak J et al., who illustrated the protective benefits of EPO in an experimental model of AKI, via multiple pathways, including reduced fibrocyte accumulation [58]. FA also in our study markedly inhibited the activation of TGF-β1/Smad signaling, as evidenced by a substantial downregulation of TGF-β1, a principal mediator of fibrosis progression. Our findings are corroborated by Ali SA et al., who asserted that FA is beneficial in inhibiting fibrosis [54]. It can prevent fibrosis by reversing the nuclear translocation of Smads and inhibiting the activation of TGF-β1/Smads signaling. Moreover, it has been shown that FA can diminish Smad3 and Smad4 by inhibiting the production of TGF-β1 and its receptor, hence mitigating fibrosis in rats [75]. According to our findings, the combination and FA-loaded Fe_3_O_4_-NPs@C treatment group showed the most significant results, indicating the synergetic effect of the antifibrotic activity of each active component.

Our data indicated that cisplatin increased Bax gene expression in renal tissues on the 7th day post-administration. Previous research stated that the Bax to Bcl-2 protein expression ratio in renal tissues increased following cisplatin induction, signifying the cells’ ability to undergo apoptosis [ndi^76^]. Alberto et al. [77] declared that after being exposed to cisplatin, it was claimed that the pro-apoptotic proteins BAX and BAK undergo structural changes that impair the integrity of the mitochondrial membrane and cause the release of apoptogenic substances such as apoptosis-inducing factor (AIF) and cytochrome C, a caspase activator. Thus, the suppression of cytochrome C, enabled by caspase activation, protected against cisplatin-induced apoptosis. In our study, the EPO-treated group exhibited a decrease in apoptotic cell death, evidenced by reduced Bax expression and elevated Bcl-2 expression. Kwak J et al. illustrate the function of EPO, which shows an anti-apoptotic effect in AKI by reducing Bax expression and maintaining Bcl-2 and Bcl-xL levels [58]. In our results, the Ferulic acid treated group exhibited notable anti-apoptotic activity, evidenced by a significant elevation in Bcl-2 levels and a marked reduction in Bax expression at the 7th of injection, corroborating prior research indicating that FA functions as an anti-apoptotic agent [11, 78] via manipulating many targets. Bcl-2 family proteins modulate mitochondrial apoptosis by altering the permeability of the outer mitochondrial membrane, initiating the subsequent caspase cascade to facilitate apoptosis [79]. Our results of Bax immunohistochemistry integrated with the expression of Bax, as in cisplatin, showed strong positive staining of Bax, which then decreased in treated groups as the level of the apoptotic marker Bax was reduced.

Ferroptosis contributes significantly to the development of AKI via triggering inflammatory responses, mostly resulting from the build-up of ferrous ions and reactive hydroxyl radicals generated by the Fenton reaction. ROS causes cellular damage, disrupts iron metabolism, and increases lipid peroxides, worsening membrane damage. Recent evidence suggests that pathways associated with Iron, lipid metabolism, and GSH play a key role in controlling the development and progression of ferroptosis. The most widely recognized pathway in the progression of ferroptosis is the glutathione peroxidase 4 (GPX4) pathway [80]. In our study, Ferroptosis was confirmed in the cisplatin group on the 7th day post-injection, as demonstrated by the reduction of GPX4, increased concentrations of MDA, signifying heightened lipid peroxidation, and the depletion of ferritin reserves due to impaired iron metabolism. According to Friedmann Angeli et al. [81] demonstrated that GPX4 deficit was shown to induce ferroptosis and AKI in mice. Another investigation showed that cisplatin induced ferroptosis in mice, as evidenced by decreased GPX4 activity, glutathione depletion, ferritinophagy-mediated free iron release, and elevated lipid peroxidation [82]. Our findings demonstrated a significant elevation in GSH and GPX4 within the FA-treated group, suggesting that FA effectively inhibited ferroptosis, consistent with prior research. This evidence highlighted FA’s role in modulating several critical factors involved in this process, including GPX4, Nrf2, and adenosine monophosphate-activated protein kinase [11].

## Conclusion

In this study, cisplatin administration induced acute kidney injury characterized by impaired renal function, anemia, oxidative stress, inflammation, and disrupted cell death pathways. The combination of EPO, FA, Fe₃O₄-NPs, and FA-loaded Fe₃O₄@C may treat AKI through its therapeutic effectiveness. This effectiveness is represented by anti-apoptotic, antioxidant, anti-inflammatory, and anti-fibrotic effects, as well as a significant ability to maintain hematological parameters. Treatment with FA-loaded Fe₃O₄@C as a drug delivery system showed the most significant therapeutic effect in our study. This effect may be due to the enhanced biocompatibility of ferulic acid provided by the nanodrug delivery system. It may also be related to the large surface area of Fe₃O₄-NPs, which promotes therapeutic effect at low dosage while reducing toxicity. This therapeutic effect against AKI is demonstrated by significantly improved renal histology, restored antioxidant status, (increased GPX4, GSH, SOD, CA and decreased MDA, reduced IL‑6 and profibrotic signaling, and normalized hematological parameters. Collectively, these findings suggest that FA-loaded Fe₃O₄@C, alone or in combination with EPO, FA, and Fe₃O₄‑NPs, is a promising nano‑therapeutic strategy for mitigating cisplatin‑induced AKI and associated anemia. This approach merits further preclinical evaluation. However, additional in vivo pharmacodynamic studies are necessary to fully understand the therapeutic potential of these nanoformulations before they are used clinically.

## Supplementary Information

Below is the link to the electronic supplementary material.

Supplementary Material 1 (DOCX 18.3 KB)

## Data Availability

Data will be available on reasonable request.
