# Supplementary material for: Erythropoietin and Ferulic Acid Loaded on Fe3O4 Nanoparticles Exert Therapeutic Effect against Acute Kidney Injury Induced by Cisplatin Via Bcl-2/Bax, IL-6, TGF-β, and GPX-4 Mechanisms
Source: Appl Biochem Biotechnol. 2026 Feb 21;198(5):3613–39. doi: 10.1007/s12010-026-05599-9 (PMC13139222; doi:10.1007/s12010-026-05599-9)
Supplement: Supplementary file 1 — Supplementary Material 1 (DOCX 18.3 KB) [file 12010_2026_5599_MOESM1_ESM.docx]

|  |  | Area | Mean | StdDev | min | max | % Area |
| --- | --- | --- | --- | --- | --- | --- | --- |
| Surface layer | Bare Surface | 681216 | 173.781 | 118.804 | 0 | 255 | 68.15 |
|  | Surface + Coating | 681216 | 172.259 | 119.385 | 0 | 255 | 67.553 |
|  | Surface + Coating + loading | 681216 | 177.654 | 117.222 | 0 | 255 | 69.668 |
|  |  | Area | Mean | StdDev | min | max | % Area |
| Pores | Bare Surface | 681216 | 158.704 | 123.623 | 0 | 255 | 62.237 |
|  | Surface + Coating | 681216 | 79.95 | 118.301 | 0 | 255 | 31.353 |
|  | Surface + Coating + loading | 681216 | 67.886 | 112.705 | 0 | 255 | 26.622 |

**Table S1:** Quantitative measurements were carried out using ImageJ/Fiji, recording mean brightness, standard deviation (StdDev), and % Area, with StdDev

The SEM analysis was performed to evaluate the effects of coating and subsequent material loading on both the surface layer and the pores of the substrate. Quantitative measurements were carried out using ImageJ/Fiji, recording mean brightness, standard deviation (StdDev), and % Area, with StdDev serving as an approximate indicator of surface heterogeneity.

For the **surface layer**, State 1 (bare) exhibited a mean brightness of 173.781, StdDev of 118.804, and % Area of 68.15%, reflecting the intrinsic texture and partial coverage of the unmodified surface. Upon application of a coating (State 2), mean brightness slightly decreased to 172.259, StdDev increased marginally to 119.385, and % Area decreased to 67.553%, indicating that the coating introduced microstructural variations while partially covering the surface. Following material loading (State 3), mean brightness increased to 177.654, StdDev slightly decreased to 117.222, and % Area increased to 69.668%, suggesting that the added material was redistributed across the surface, partially smoothing irregularities and slightly increasing surface coverage.

For the **pores**, State 1 (bare) had a mean brightness of 158.704, StdDev of 123.623, and % Area of 62.237%, indicating open or partially filled pores with high heterogeneity. In State 2 (coated), mean brightness decreased significantly to 79.950, StdDev slightly decreased to 118.301, and % Area dropped to 31.353%, demonstrating that the coating partially filled the pores, reducing both visible pore area and heterogeneity. After loading material on the coated surface (State 3), mean brightness further decreased to 67.886, StdDev decreased to 112.705, and % Area dropped to 26.622%, indicating additional pore filling and a more homogenized internal structure.

Overall, these results confirm that surface coatings modify the surface texture while partially covering the surface, and subsequent loading progressively fills the pores, reducing heterogeneity and altering both brightness and coverage. The combined analysis of surface layer and pores provides clear evidence that the added layers effectively modify both external and internal features of the substrate.

**References**

1. Synthesis of Silica-Coated Fe_3_O_4_ Nanoparticles by Microemulsion Method: Characterization and Evaluation of Antimicrobial Activity
2. Modification and Characterization of Fe3O4 Nanoparticles for Use in Adsorption of Alkaloids
3. Preparation of Fe3O4/vineshoots derived activated carbonnanocomposite for improvedremoval of Cr(VI) from aqueoussolutions
4. Facile Preparation of Fe3O4/C Nanocomposite and Its Application for Cost-Effective and Sensitive Detection of Tryptophan
